# Hemagglutinin 222 Variants in Pandemic (H1N1) 2009 Virus

**DOI:** 10.3201/eid1704.100784

**Published:** 2011-04

**Authors:** Maria Beatrice Valli, Marina Selleri, Silvia Meschi, Paola Zaccaro, Donatella Vincenti, Eleonora Lalle, Maria Rosaria Capobianchi, Stefano Menzo

**Affiliations:** Author affiliation: National Institute for Infectious Diseases, Virology, Rome, Italy

**Keywords:** Pandemic, influenza, viruses, hemagglutinin, evolution, D222G, letter

**To the Editor:** The biologic role of amino acid variants at position 222 of the hemagglutinin (HA) gene of pandemic (H1N1) 2009 virus in severe infections has been extensively discussed. A recent series of studies (*1**–**3*) confirm the initial suggestions that G or N in this position might confer greater pathogenic potential to the virus than to the wild type. In contrast, their data suggest that no particular pathogenicity is associated with the 222E variant because it occurs at the same frequency in severe and mild infections. Most authors also seem to agree that D222G or N appears sporadically in phylogenetically distant viruses, with limited transmissibility.

However, Puzelli et al. (*4*) reported transmission of a 222G mutant from son to father (with the appearance of an additional G155E mutation). In Italy, the pattern of D222 variants has been peculiar, with extremely rare appearances of 222G and high diffusion of 222E isolates. At the National Institute for Infectious Diseases in Rome, 82 isolates (GenBank accession nos. CY063455–CY063469 for new sequences in this study) were monitored for D222 variants. No 222G or N variants were detected, even in 24 severe infections, nor was the G155E mutation detected. This finding was not surprising, given the worldwide low frequency of this mutation, even in severe infections.

Conversely, D222E was detected in 12 of the 82 cases, peaking in September 2009, when it was present in most of the infections, with no overrepresentation in severe cases. Subsequently, it was substituted by different 222D viruses during the autumn–winter outbreak. The analysis of publicly available sequences from other centers in Italy confirmed the trend: 222E was the dominant variant during the summer, 222G was detected only in 4 cases, and 222N was never detected. As we previously reported (*5*), phylogenetic analysis of 222E variants allowed identification of them as an authentic circulating subclade of clade 7 (*6*) or cluster 2 (*7*), rather than as sporadically occurring variants.

To further investigate the origin and the evolution of 222 variants, we have extended the phylogenetic analysis (neighbor-joining) to 2,492 complete HA sequences from the Global Initiative on Sharing All Influenza Data database (expanded Figure online, www.cdc.gov/EID/content/17/4/749-F.htm), confirming the clustering of all the worldwide 222E variants in a well-defined subclade (Figure; expanded Figure online). The same analysis showed that D222G variants could be reconciled with 2 different phylogenetic patterns. The first less frequent pattern includes sequences appearing sparsely throughout the tree, confirming the mentioned hypothesis of sporadic mutation. In contrast, the second pattern (the majority) relates to small groups of sequences appearing in monophyletic microclusters. Among these microclusters, 2 are particularly interesting because they include only 222G sequences, isolated in different parts of the world (Figure). This finding is still compatible with sporadic mutation; bootstrap values are low because of the low general variability of these sequences. However, the possibility that D222G variants are transmissible and might sustain small epidemics of their own or that they might arise more easily from specific, phylogenetically related backgrounds, is intriguing. In a few countries, such as Italy, Norway, or Sweden, where the 222E virus has been circulating as a substantial proportion of the total virus, the 222G variants appeared more frequently in the genetic context of the 222E virus (*1**,**4*), as demonstrated by phylogenetic analysis and confirmed by the analysis of codon 239 (the codon determining the 222 residue specificity): GAA to GGA (E to G), instead of GAT to GGT (D to G). In these cases, the correct definition of 222G variants would therefore be E222G rather than D222G. From this point of view, the 222N variant would have a higher genetic barrier to change from E because it would require 2 mutations (GAA to AAT) instead of 1 (GAT to AAT), and indeed none of the 16 available (worldwide) 222N full-length variants clustered with the 222E virus. On the basis of these findings, 2 different amino acids, D and E, might be considered polymorphic variants at position 222, and the potentially more pathogenic mutants or circulating variants would be G or N.

**Figure:**
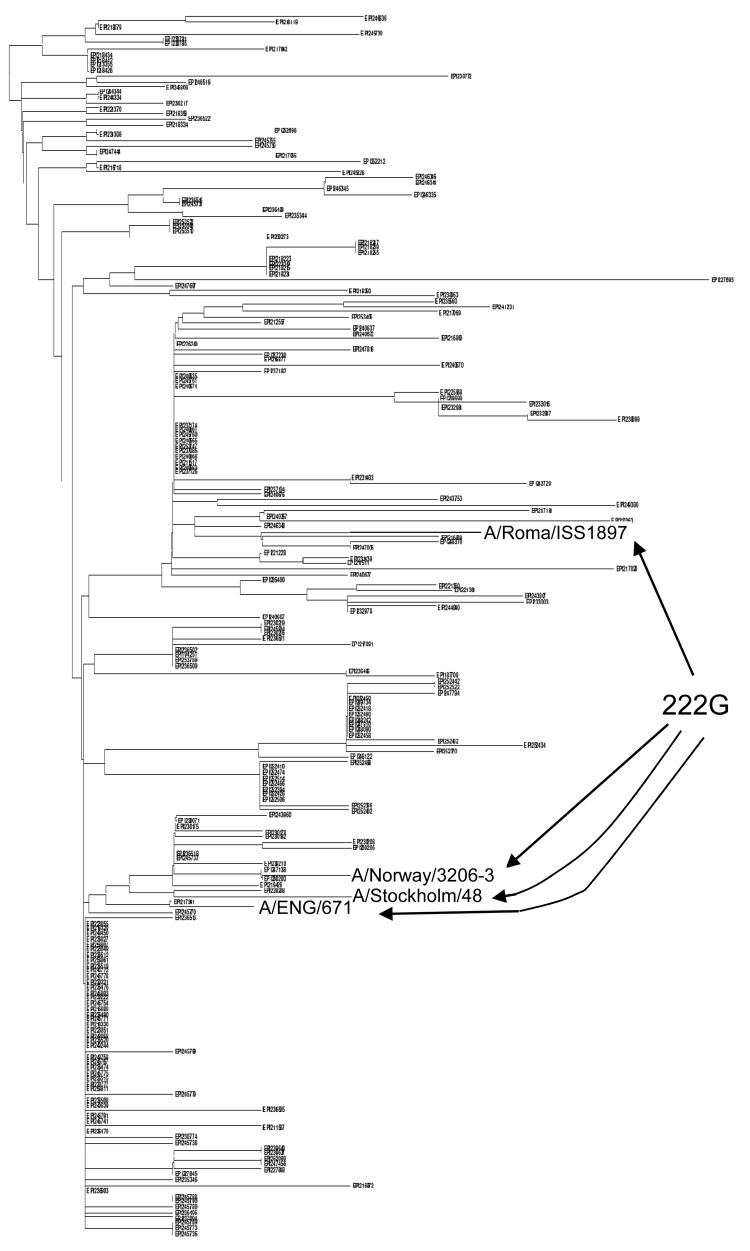
Phylogenetic relationships among 2,492 complete hemagglutinin (HA) genes of pandemic (H1N1) 2009. At the center, the whole neighbor-joining tree. On the left, enlargement of 2 regions of the tree including pure monophyletic D222G clusters, indicated in blue. On the right, enlargement of the monophyletic D222E virus cluster, including 98% of the global 222E isolates (red box). E222G variant isolates, as examples, respectively, from Italy (*4*), Norway (*1*), Sweden, and the United Kingdom, are indicated in blue. The sequence labels represent the Global Initiative on Sharing All Influenza Data serial numbers; those of particular interest for this study are indicated by the strain name or country of origin.
